# Creating a gold medal Olympic and Paralympics health care team: a satisfaction survey of the mobile medical unit/polyclinic team training for the Vancouver 2010 winter games

**DOI:** 10.1186/1756-0500-6-462

**Published:** 2013-11-13

**Authors:** D Ross Brown, Behrouz Heidary, Nathaniel Bell, Leanne Appleton, Richard K Simons, David C Evans, S Morad Hameed, Jack Taunton, Kosar Khwaja, Michael O’Connor, Naisan Garraway, Peter Hennecke, Donna Kuipers, Tracey Taulu, Lori Quinn

**Affiliations:** 1 Trauma Services, Vancouver General Hospital, 855 West 12th Avenue, Vancouver, British Columbia, V5Z 1 M9, Canada; 2 Department of Surgery, University of British Columbia, Vancouver, Canada; 3 Provincial Health Services Authority, Vancouver, Canada; 4 Vancouver Coastal Health, Vancouver, Canada; 5 Division of Sports Medicine, Faculty of Medicine, University of British Columbia, Vancouver, Canada; 6 Trauma Services, McGill University Health Center, Montreal, Canada; 7 Department of Emergency Medicine, University, Kingston, Ontario, Canada; 8 College of Nursing, University of South Carolina, Columbia, USA

**Keywords:** Mobile medical unit, Medical education, Curriculum, 2010 Vancouver Olympic Winter Games

## Abstract

**Background:**

The mobile medical unit/polyclinic (MMU/PC) was an essential part of the medical services to support ill or injured Olympic or Paralympics family during the 2010 Olympic and Paralympics winter games. The objective of this study was to survey the satisfaction of the clinical staff that completed the training programs prior to deployment to the MMU.

**Methods:**

Medical personnel who participated in at least one of the four training programs, including (1) week-end sessions; (2) web-based modules; (3) just-in-time training; and (4) daily simulation exercises were invited to participate in a web-based survey and comment on their level of satisfaction with training program.

**Results:**

A total of 64 (out of 94 who were invited) physicians, nurses and respiratory therapists completed the survey. All participants reported favorably that the MMU/PC training positively impacted their knowledge, skills and team functions while deployed at the MMU/PC during the 2010 Olympic Games. However, components of the training program were valued differently depending on clinical job title, years of experience, and prior experience in large scale events. Respondents with little or no experience working in large scale events (45%) rated daily simulations as the most valuable component of the training program for strengthening competencies and knowledge in clinical skills for working in large scale events.

**Conclusion:**

The multi-phase MMU/PC training was found to be beneficial for preparing the medical team for the 2010 Winter Games. In particular this survey demonstrates the effectiveness of simulation training programs on teamwork competencies in ad hoc groups.

## Background

Mobile medical units often serve as extensions of main health facilities to reach patients when they are at most risk. These high-tech units act as mini-hospitals to provide definitive life-saving emergency and/or post disaster response for many needs-based functions, including disaster response, large-scale recreational events, support in mass casualties, or replacing lost ambulatory or emergency room service capacity in case of emergency department closures [1-5].

During the 2010 Vancouver Winter Games, mobile surgical services and medical support for athletes and officials were provided on-site at the Whistler Athlete Village by the Mobile Medical Unit/Polyclinic (MMU/PC). The MMU/PC was designed to provide definitive life-saving care for persons in the event that injury severity, transportation, or weather disruption would prohibit immediate triage and transport to larger care facilities as well as to provide surge capacity in the event of a mass or multi-casualty situation.

**Figure 1:**
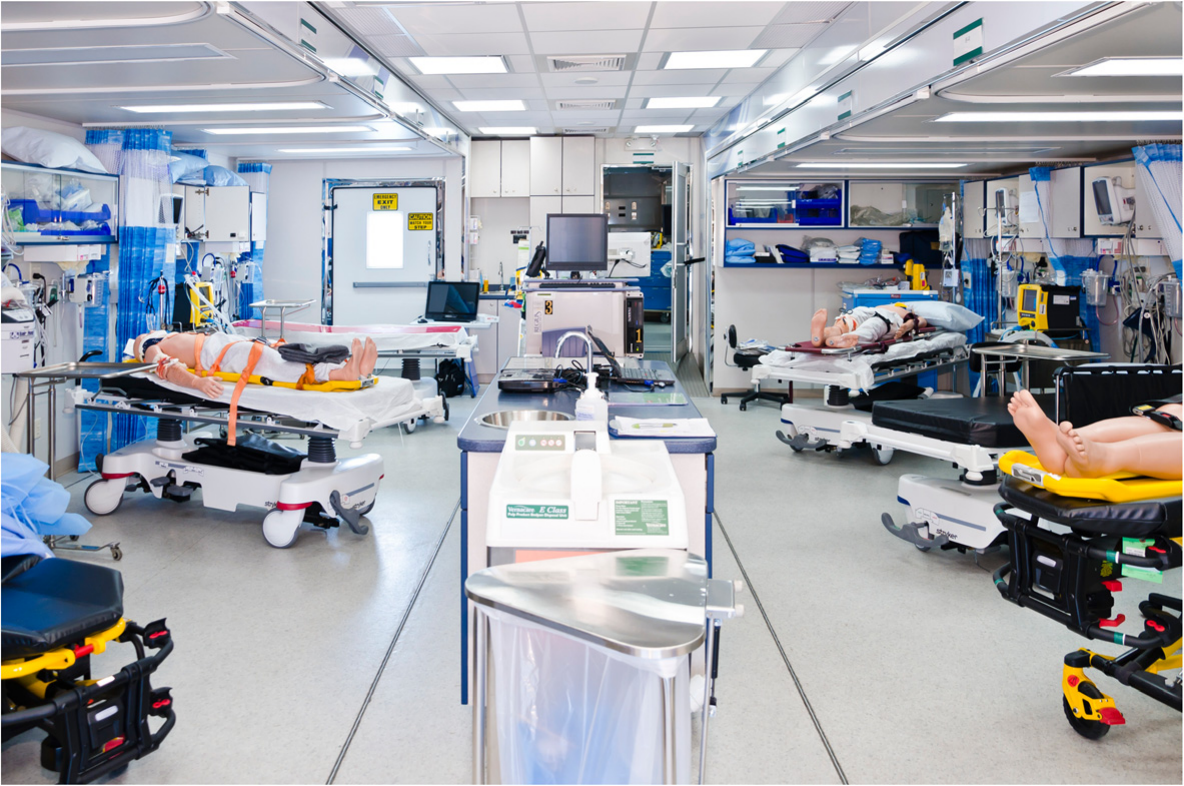
The MMU provided critical and surgical care capability in the Whilster Athletics Village during the 2010 Olympic and Paralympic Winter Games.

**Figure 2:**
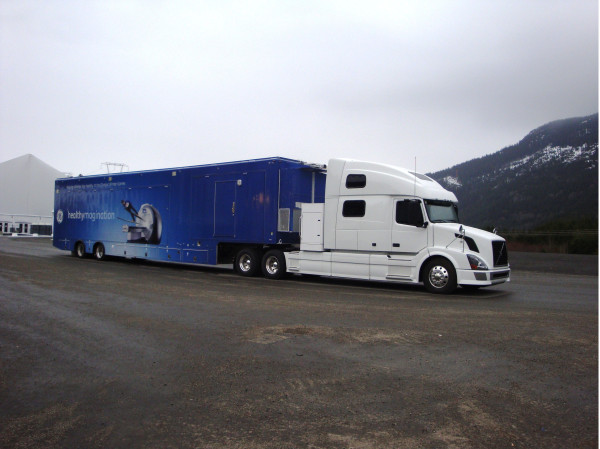
**The Mobile Medical Unit in transport configuration (additional images and information can be found at: **http://www.bcmmu.ca/default.htm**).**

The MMU is a 15.9-metre tractor-trailer, which can expand to a 90-square-metre unit with up to 12 beds (Figures 1 and 2). Our configuration included four resuscitation bay/critical care beds, a single table operating room and two non-monitored holding beds. The unit was supported with a secondary trailer stocked with 72 hours worth of medical/surgical supplies and other equipment. The MMU had self-contained back up diesel generators, an O_2_ concentrator, IMIT connectivity, and lab and diagnostic support services.

As these are highly sophisticated and specialized units it is necessary to provide equally sophisticated and specialized training in order to make the users comfortable and familiar with the environment. It has been shown that simulation based trainings have demonstrated significant impact on teamwork competencies such as communication, role clarity, situation awareness and leadership as well as clinical knowledge and skills [6,7]. An education framework was established early in the planning process to ensure all medical staff and contractors received hands-on training to prepare for efficient and effective patient care and flow while working in the MMU. This training program was coordinated by a joint education committee between Canadian Forces Trauma Training Centre (West) (CFTTC (W))^a^ and the Vancouver Coastal Health (VCH) section of Learning and Development at Vancouver General Hospital (VGH) with close collaboration from the Public Health Agency of Canada (PHAC), National Office of Health Emergency Response Teams (NOHERT) and Vancouver Olympic Committee (VANOC).

Validation and participant satisfaction studies can help link field performance to training context [8,9]. The feedback gained from these measures can be used to synchronize the training objectives with trainees’ area of expertise to make the training “more targeted” [10]. The purpose of this study was to survey the satisfaction of the clinical staff that completed the MMU/PC training in attempt to assess the impact of the education and training program provided to health care providers who supported the MMU during the Games.

## Methods

### Participants

A total of 105 medical personnel volunteers and contractors were recruited from within the Province of British Columbia and from across Canada to provide the required medical expertise to staff and operate the MMU during the 2010 Winter Games. Recruitment advertisements specified qualifications/criteria for selection. Successful applicants were credentialed through VGH, VCH, and VANOC prior to enrollment. Experts recruited for participation included anesthesiologists, general and orthopaedic trauma surgeons, emergency, critical care, and operating room nurses, and respiratory therapists.

Participants were grouped into five 17-person teams. Each team comprised of two trauma/general surgeons, two anesthesiologists, two orthopedic trauma surgeons, four operating room nurses, four critical care nurses, two emergency nurses, and one respiratory therapist. Each team was deployed for a 10 to 14 day rotation to provide care and continued support for the Whistler Olympic Village Polyclinic.

### Education and training model

MMU participants completed a four-phase education and training curriculum prior to deployment in effort to foster the performance of a high functioning interdisciplinary team. Instruction was provided in collaboration with VGH, CFTTC(W), the Centre for Excellence for Simulation Education and Innovation (CESEI) at VGH, and the VCH Learning & Development division. Training programs were delivered between October 2009 and March 2010. The training model included the following phases:

#### Phase 1: weekend training

In October 2009, medical personnel attended a 2.5 day workshop at VGH. The purpose of this training phase was to introduce team members to other participants and provide an opportunity to become accustomed to the MMU facility. During this time all participants were also orientated with the triage and delivery structure for how medical services were to be provided across all Olympic venues. As well they were provided with classroom learning and instruction to increase their content familiarity with the MMU in order to problem solve potential treatment challenges they may face.

#### Phase 2: web-based modules

Following the weekend training seminar, participants completed a series of “mandatory” and elective web-based learning modules. Learning modules were distributed through CESEI and VCH Learning and Development. Topics included Infection Control Basics (hand hygiene), Central Venous Catheter Care & Maintenance, Safe Blood Transfusion, Workplace Hazardous Materials Information System Basics, VANOC 2010 medical services, Introduction to the Mobile Medical Unit – History and Planning, Summary of Whistler Polyclinic and Mobile Medical Unit, Mobile Medical Unit Orientation to Physical Lay Out and Patient Flow, 2010 Olympic/Paralympic MMU Blood Education Overview, Transfusion Medicine Services, Introduction to METI Emergency Care Simulator (ECS) and Introduction to METI Human Patient Simulator (HPS). Mandatory learning modules included Blood Transfusion and Infection Control Basics (hand hygiene).

#### Phase 3: “just-in time” training

In the week prior to deployment, participants completed a series of simulation training exercises in a mock up MMU in CESEI over the span of 1.5 days. Training included simulations as well as small and large group sessions. CFTTC(W) provided leadership in each exercise. Simulation exercises escalated in complexity over time, beginning with: (1) an introduction of team dynamics and trauma protocols; (2) orientation session and familiarization with the mock trauma bay, OR, Emergency Care Simulator (ECS) and Human Patient Simulator (HPS); (3) scenario based trauma management sessions followed by debriefings; and (4) mass casualty and complex case-based scenarios followed by debriefings. Simulation exercises were modified based on scenarios learned from previous team experiences consistent with a rapid cycle change problem solving philosophy.

#### Phase 4: daily simulation training

Simulation and training were imbedded into the daily routines for the MMU and polyclinic team. Daily simulation exercises ranged in complexity, beginning with simple case scenarios and moving toward more complex situations such as Code Blue simulations in all areas of the Whistler Polyclinic (eg, Dentistry, Therapy, MRI), a series of outreach responses in the Whistler Athletes Village, and the complex Long-Line Helicopter Evacuation from the event scene to the MMU. Arrangements were made to run these simulations while still conducting the normal operations of the Polyclinic. All scenarios were executed in “real time” whenever possible with fully integrated communications with other participating agencies (eg, event security, ski patrol etc.). An important aspect of the training was the formative debriefings held after each simulation. The debriefings allowed learnings to be discussed that built further team confidence within the new environment.

### Survey of clinical staff satisfaction of the training program

Clinical staff were recruited by e-mail using their contact information obtained from the MMU/PC management office database. Inclusion criteria for our study were as follows: (1) having completed a rotation of at least four days serving with the MMU/PC in Whistler during the Games, and (2) having completed at least one of the four phases of pre-deployment training. All persons who participated in the preparation or delivery of the MMU/PC training programs were excluded from the survey.

Participants received a letter of initial contact from the Principal Investigator outlining the purpose and procedures of the study. Attached to this email they received a link to a secure online survey hosted at FluidSurveys (Chide.it Inc., Ottawa, Ontario).

### Training assessment survey

All assessment questions were constructed from focus group discussions with MMU/PC staff. Questions were based on the phases of training that were provided prior to deployment and pilot-tested with MMU/PC staff. The survey was divided into three sections: responses to individual training phases; responses on overall course satisfaction; and demographic and work-related experience profiles. The survey was designed to require approximately 15 minutes to complete and would allow participants to save their progress in the event that the could not complete the entire survey in one sitting.

Most of the questions were closed-ended, but participants were encouraged to provide additional content in the event they wished to provide more specific feedback. Close-ended questions followed a five-point Likert scale ranging from very valuable, somewhat valuable, neutral, minimally valuable, and not at all valuable. The survey also included open-ended questions in which participants were asked to respond in their own words regarding the quality and amount of simulation training that was provided, whether they have since implemented or incorporated any of the learning approaches into practice at their home hospital/agency, and whether they had any other comments or suggestions regarding the education and training that they received during the MMU/PC training period. The survey was posted on-line between May 20, 2011 and August 8, 2011. The study was approved by the Behavioral Research Ethics Board at the University of British Columbia.

### Statistical assessment

Descriptive statistics and cross-tabulations were calculated for each survey item. Responses were stratified by clinical job title, years of experience, and prior experience working in large scale events. Differences between means of continuous variables were examined using a two-tailed t-test, and differences in proportions of categorical variables were examined using a chi square test. We examined all categorical variables where expected values were less than five using Fisher’s exact test. A significance level of 0.05 was used to assess all bivariate relationships. All statistical analyses were generated using SAS software, Version 9.2 for Windows [11].

## Results

Of the 94 participants that were contacted, 64 completed the on-line survey (68%). The average age of participants was 44 years (range 28 to 64). Table 1 lists the study population by clinical role. The average number of years of prior clinical experience for all participants was 15 years (range 0.5 – 39). The percentage of participants with prior experience working at large scale events was 55%. A total of 11 participants had previous military training in health care. All personnel with military training had pervious experience working in large-scale events, which included conflict and disaster response tours in Bosnia-Herzegovina, Afghanistan, and Haiti. Participants were similar in years of clinical work experience (χ^2^ **=** 0.0703, p = 0.791, df = 1), but their characteristics varied when comparing the number of participants that had prior experience working in large scale events (χ^2^ = 7.4006, p = 0.006, df = 1) (Table 2).

**Table 1:** Clinical profile of participants

**Clinical role**	**N**	**%**
Nurses and RT’s*	*34*	*53.1*
Critical care nurse	14	21.9
Emergency nurse	6	9.4
Operating room nurse	10	15.6
Respiratory therapist	4	6.3
*Physician*	*30*	*46.9*
Anesthesiologist	12	18.8
Emergency physician	1	1.6
Orthopedic surgeon	6	9.4
Trauma surgeon	11	17.2

*Respiratory Therapists.

**Table 2:** Clinical experience profile

**Clinical experience**	Nurses and RT’s*	**Physicians**	**P value**
***Overall work experience***			0.791
< 5 years	8	6	
> 5 years	26	24	
***Large scale work experience***			0.006
No experience	10	19	
Prior experience	24	11	

*Respiratory Therapists.

### Training phase 1: weekend training

Summary statistics for the *weekend training* are listed in Table 3. On average, all participants rated the training curriculum favorably (average curriculum score: 1.70). Areas of least interest by all participants included discussion on daily routines, shifts, and schedules [nursing and RT response average: 2.00 (SD 1.05); physician staff responses: 2.36 (SD 1.05)] and discussion section on uniforms, security, and accommodation [nursing and RT staff response average: 1.86 (SD 1.11); physician response average: 2.04 (SD 1.09)]. No significant differences in response patterns between nursing and RT staff and physician staff were observed. Responses to the entire weekend training curriculum were similar among clinical personnel when contrasted against years of work experience and among respondents having previous training in large scale events (table not shown).

**Table 3:** Responses to the weekend training session among nursing and physician course participants

		Average response^*^	**Average responses by clinical role**		
**No.**	**Questions**	**All participants**	**Nurses and RT’s**^§^**n = 21 (SD)**	**Physicians n = 22 (SD)**	**P value**
1	Training valuable for experiences during the games	1.43 (0.63)	1.43 (0.75)	1.43 (0.51)	0.520
2	Training increased comfort and familiarity in work environment	1.48 (0.50)	1.43 (0.51)	1.54 (0.51)	0.547
3	Preparation for subsequent practice during the games	1.56 (0.55)	1.52 (0.60)	1.60 (0.50)	0.437
4	MMU ’walk through’ provided situational awareness	1.27 (0.59)	1.24 (0.43)	1.32 (0.72)	1.000
5	MMU ’walk through’ increase anxiety	4.02 (1.01)	3.9 (1.04)	4.13 (0.99)	0.455
6	Discussion seminar on medical liability	1.78 (0.69)	1.67 (0.73)	1.90 (0.64)	0.356
7	Discussion on care management of elite athletes	1.63 (0.80)	1.48 (0.60)	1.80 (0.95)	0.288
8	MMU ’walk through’: understanding role and procedures	1.46 (0.67)	1.57 (0.81)	1.36 (0.49)	0.809
9	MMU ’walk through’: movement of simulated patient	1.45 (0.63)	1.50 (0.69)	1.41 (0.59)	0.896
10	MMU ’walk through’: experience in physical layout	1.38 (0.58)	1.40 (0.60)	1.36 (0.58)	1.000
11	Discussion on uniforms, security, and accommodation	1.95 (1.09)	1.86 (1.11)	2.04 (1.09)	0.793
12	Discussion on daily routines, shifts, and schedules	2.19 (1.05)	2.00 (1.05)	2.36 (1.05)	0.334
13	Discussion on protocols, safety, and infection control	1.72 (0.88)	1.67 (0.66)	1.77 (1.07)	0.199
14	General Q & A discussion	1.69 (0.75)	1.52 (0.68)	1.86 (0.79)	0.357
15	Discussion on mass casualty and emergency planning	1.72 (0.70)	1.57 (0.68)	1.86 (0.71)	0.408
16	Discussion on pandemic planning and H1N1	1.86 (0.83)	1.71 (0.64)	2.00 (0.97)	0.730
17	Discussion on blood administration protocols	1.76 (0.96)	1.42 (0.51)	2.09 (1.18)	0.109
18	Discussion on patient transfer protocols	1.58 (0.76)	1.57 (0.51)	1.59 (0.96)	0.215

^*§*^Respiratory Therapists, *Response scoring scale: 1 = very valuable, 2 = somewhat valuable, 3 = neutral, 4 = minimally valuable, 5 = not at all valuable.

### Training phase 2: web-based training

Summary statistics for the *web-based training* are listed in Table 4. On average all participants rated the web-based training favorably, although less favorable than the Phase 1 weekend training (average web-based training score: 2.04). When compared against clinical role, nurses and RT’s reported more favorably to Safe Blood Transfusion [1.89 (SD 0.99) vs. 2.42 (1.07); p 0.043], ECS training [1.68 vs. 2.50; p 0.007], and HPS training [1.82 vs. 2.38; p 0.008].

**Table 4:** Responses to the web-based training seminar among nursing and physician course participants

		Average response^*^	**Average responses by clinical role**		
**No.**	**Questions**	**All participants**	**Nurses and RT’s**^§^**n = 21 (SD)**	**Physicians n = 22 (SD)**	**P value**
1	Infection control basics	2.31 (1.01)	1.93 (0.70)	2.65 (1.12)	0.054
2	Central venous catheters care & maintenance	2.44 (1.16)	2.08 (0.91)	2.75 (1.27)	0.296
3	Safe blood transfusion	2.16 (1.08)	1.89 (0.99)	2.42 (1.07)	0.043
4	WHMIS basics	2.35 (0.97)	2.18 (0.98)	2.52 (0.95)	0.254
5	VANOC medical services	1.85 (0.72)	1.81 (0.79)	1.89 (0.67)	0.416
6	Introduction to MMU: history and planning	1.71 (0.75)	1.61 (0.75)	1.80 (0.76)	0.315
7	Summary of Whistler polyclinic and MMU	1.66 (0.76)	1.57 (0.69)	1.75 (0.83)	0.312
8	MMU orientation to physical layout and patient flow	1.76 (0.90)	1.81 (0.79)	1.71 (1.01)	0.615
9	Olympics/paralympics MMU blood education overview	2.07 (1.04)	1.68 (0.72)	2.46 (1.17)	0.075
10	Transfusion medicine services	2.07 (0.84)	1.81 (0.78)	2.31 (0.85)	0.171
11	Introduction to METI Emergency Care Simulator (ECS)	2.06 (1.03)	1.68 (0.80)	2.50 (1.10)	0.007
12	Introduction to METI Human Patient Simulator (HPS)	2.10 (0.96)	1.77 (0.81)	2.48 (0.99)	0.008
13	Value in post-test assessments of learning modules	2.09 (0.98)	1.82 (0.81)	2.38 (1.06)	0.329

^*§*^Respiratory Therapists, *Response scoring scale: 1 = very valuable, 2 = somewhat valuable, 3 = neutral, 4 = minimally valuable, 5 = not at all valuable.

### Training phase 3: just-in-time training

On average all participants rated the just-in-time training phase favorably (average score excluding blood bank session: 1.59). Comparison statistics for the *just-in-time MMU training* are listed in Table 5. When compared against clinical role, nurses and RT’s reported more favorably to the simulation training that emphasized competencies and knowledge in clinical skills [1.44 (SD 0.58) vs. 2.56 (1.23); p 0.002]. On average, respondents without past experience working in large scale events similarly responded more favorably to the simulation training exercises that emphasized competencies and knowledge in clinical skills [1.60 (SD 0.87) vs. 2.33 (SD 1.18); p 0.009]. Responses to the just-in-time training curriculum were similar among clinical personnel when contrasted against years of work experience.

**Table 5:** Responses to the just-in-time MMU training seminar among nursing and physician course participants

		Average response^*^	**Average responses by clinical role**		
**No.**	**Questions**	**All participants**	**Nurses and RT’s**^**§ **^**n = 21 (SD)**	**Physicians n = 22 (SD)**	**P value**
1	Effectiveness of the introductory session	1.46 (0.58)	1.52 (0.58)	1.40 (0.58)	0.694
2	Simulation training: competency & communication	1.40 (0.80)	1.41 (0.69)	1.40 (0.92)	0.344
3	Simulation training: knowledge	1.62 (0.89)	1.30 (0.48)	1.95 (1.12)	0.075
4	Simulation training: clinical skills	1.98 (1.09)	1.44 (0.58)	2.56 (1.23)	0.002
5	Value of simulation training for weekend training session	1.45 (0.77)	1.38 (0.64)	1.54 (0.91)	0.283
6	Value of simulation training for web-based training session	1.67 (0.99)	1.63 (0.97)	1.71 (1.04)	0.525
7	Value of blood bank training session^†^	1.65 (0.89)	--	--	--

^*§*^Respiratory Therapists, *Response scoring scale: 1 = very valuable, 2 = somewhat valuable, 3 = neutral, 4 = minimally valuable, 5 = not at all valuable,^*†*^*Course provided for nursing staff only.*

### Training phase 4: daily simulation training

Summary statistics for the *daily simulation training* are listed in Table 6. On average the daily simulation training phase was rated by all participants as the most favorable phase (average score: 1.24). When compared against clinical role, neither nurses and RT’s nor physicians differed in their responses to the simulation training curriculum, with both groups reporting favorable experiences on each of the three simulation components conducted during the MMU/PC training period. No differences in response feedback were observed among participants with past experience working in large scale events or among clinical personnel with different years of work experience.

**Table 6:** Responses to the daily simulation training sessions among nursing and physician course

		Average response^*^	**Average responses by clinical role**		
**No.**	**Questions**	**All participants**	**Nurses and RT’s**^§^**n = 21 (SD)**	**Physicians n = 22 (SD)**	**P value**
1	Patient moving simulation exercises	1.20 (0.41)	1.21 (0.42)	1.18 (0.39)	0.759
2	Long line helicopter simulation exercises	1.28 (0.71)	1.15 (0.36)	1.39 (0.90)	0.361
3	Simulation debriefing sessions	1.24 (0.62)	1.17 (0.38)	1.30 (0.77)	1.000

^*§*^Respiratory Therapists, *Response scoring scale: 1 = very valuable, 2 = somewhat valuable, 3 = neutral, 4 = minimally valuable, 5 = not at all valuable.

### Open-ended responses

On average, both groups responded favorably to the simulation training. Approximately 55% of physicians (n = 10) who elected to provided feedback felt that they received adequate simulation training prior to deployment, while 39% of physicians (n = 7) recommended that similar training schedules should be increased if the course were to be offered again. The most common rationale for providing more simulation training among physicians was to improve familiarity working in the MMU unit. Among nurses and RT’s who elected to comment on the quality and amount of simulation training, 44% (n = 7) felt that the quality and amount of training was sufficient while 47% (n = 9) suggested that more simulation training would have been beneficial. The most common rationale for providing more simulation training among nurses and RT’s was to improve familiarization and movement within the MMU.

Only 5 of the 34 nursing and RT staff participants elected to provide additional feedback regarding whether they had implemented or incorporated any of the learning approaches into practice at their home hospital/agency. Of those responses, each participant reported favorably that the training has positively impacted their daily routine, but did not elect to comment on a particular component of the training that they found to be beneficial. One individual reported that the MMU/PC simulation training has improved their team debriefing following regular simulator training sessions at their own hospital/agency. Two individuals reported that they now regularly incorporate simulation training with their students/staff as a result of the training provided at the MMU/PC.

## Discussion

To assess the applicability of medical training in the actual practice field, the impact of training should be questioned in several territories such as clinical skills, performing procedures, patient management, ethicolegal responsibilities, team performance and communication skills [12,13]. Our multi-phase MMU/PC training, that covered a diverse array of areas, was found to be beneficial in the perspective of the participants for preparing personnel for the 2010 Winter Games. The uniqueness of this training program was a four-phase education and training curriculum designed to foster the performance of a high functioning interdisciplinary team.

Although our evaluation did not include a controlled clinical trial design, it provides valuable information on the perspective of clinical staff that completed the different training models developed for the curriculum. In particular this survey demonstrates strong support for incorporating simulation training when preparing ad hoc clinical training programs. These findings add additional support to the literature on the utility of simulation training programs on teamwork competencies [14]. The study also provides new information on how to structure training programs for preparing clinicians to work within mobile medical environments.

We had a favorable response rate and good representation of among the clinical professionals who participated in this training program. Over 75% of the participants had more than 5 years work experience and 55% had experience working in large scale events, indicating that the Whistler Olympic Village recruitment strategy specifically targeted individuals with experience working in large-scale events and experience in trauma. The fact that the ‘more experienced’ groups rated the training favorably does provide an indication that the structure of the training program was useful and of benefit to the participants.

This feedback is important given the many potential situations where ad hoc clinical teams might form, from potential epidemic outbreaks, to terrorist threats, to natural disasters. Since the 2010 Games, daily simulation has become a standing operation procedure for current MMU deployments in effort to keep our new and constantly changing teams up to speed on working in these types of clinical environments. Subsequent deployments have ranged from staging at small stage events, including the BMX Supercross World Cup in Abbotsford, BC in 2012, to outreach programs across the province, to providing Emergency Department support over a 10-day period during the 2012 flooding of the Surrey Memorial Hospital. Many of the original teaching staff attached to the MMU during the 2010 Games subsequently became staff for the MMU as a provincial resource. We continue to build on the original ‘just in time’ training curriculum to help train and prepare the medical teams prior to deployment. These ongoing sessions are helping to re-shape the curriculum, taking lessons learned from previous deployments and immediately updating lesson plans and performance objectives.

At the time of the Games, we had little experience in the MMU. Knowing what we do now about the strengths and weaknesses of the unit and support trailer and from experience with the Games and other deployments we would recommend holding more simulation exercises of casualties for future training sessions. In addition, to optimize learning for all clinical staff we would have the MMU wired for video broadcast of simulations. We recently did this at an emergency medical conference, allowing hundreds of people to watch the small teams function in the unit. For education and training purposes, the benefit of the video stream would be the increased observation opportunities for clinical staff as they watched teams practice, thus affording another opportunity to gain experience for working within the MMU.

These results should be viewed within the context of some important limitations. Firstly, although the survey responses imply that the training program developed for the 2010 Games was effective, we do not present any clinical outcome data that can additionally confirm or deny the appropriateness of the training. Access to this data was not feasible due to ownership and privacy stipulations of the medical records for all persons treated in the MMU during the 2010 games. One approach for future training evaluation programs would be to hold pre and post training surveys, thereby linking the training and performance objectives to outcome measures such as patient satisfaction, complications, or functional outcomes. Similarly, future assessments could be derived using pre- and post-test knowledge and confidence tests and surveys followed by post-deployment validation surveys. For example, an external reviewer observing team performance for error rates with simulation patients and treatment algorithms.

## Conclusion

One of the legacy goals of the MMU/PC is to maintain an annual clinical training program whereby portions of the training delivered during the 2010 Winter Games will be replicated. Within a Canadian context, British Columbia is the only province with this type of service facility. Thus, it offers an opportunity to improve the preparedness of Canadian clinical personnel who may later work in austere environments either in Canada or abroad. The level of satisfaction from participants of the training program is encouraging as it provides a foundation to structure future training clinical training programs for working in MMU environments.

## Endnote

^a^CFTTC(W) contribution with the permission of the Minister of National Defense.

## Competing interests

DRB, LA, and PH were employed by the British Columbia Health Authority to staff the MMU during the 2010 Olympic Winter Games. All other authors declare that they have no competing interest.

## Authors’ contributions

DRB conceptualized the idea for this study and led in the drafting and development of the manuscript. BH and NB conducted the statistical analyses, created the tables, and drafted an early version of the manuscript. All authors participated in the drafting of the survey questions and in the writing of the initial and final versions of the manuscript.
